# Analysis of the Anti-inflammatory Effects of a Delivery System Based on Alginate Hydrogels Enriched with Bioactive Compounds from Marine Sponge *Dysidea robusta*

**DOI:** 10.1007/s12010-026-05684-z

**Published:** 2026-04-06

**Authors:** Homero Garcia-Motta, Cintia Cristina Santi Martignago, Mirian Bonifacio, Dalete Christine da Silva Souza, Lais Caroline Souza-Silva, Beatriz Soares-Silva, Anabella Patricia Rosso, Isabelly Bertochi Veroneze, Marcelo Assis, Fernando Carlos Giacomelli, Sandra Andrea Cruz, João Henrique Ghilardi Lago, Renata Granito, Lívia Assis, Alessandra Mussi Ribeiro, Ana Claudia Muniz Rennó

**Affiliations:** 1 Department of Biosciences, Federal University of São Paulo (UNIFESP), Silva Jardim Street, 136, Santos, SP 11015020 Brazil; 2 Center for Natural and Human Sciences, Federal University of ABC (UFABC), Av. dos Estados, 5001 - Bangú, Santo André, SP 09280-560 Brazil; 3 Chemistry Department, Federal University of São Carlos (UFSCar), Rod. Washington Luís, s/n - Monjolinho, São Carlos, SP 13565-905 Brazil; 4 Scientific Institute and Technological Department - University Brazil, Itaquera, São Paulo, SP 08230-030 Brazil

**Keywords:** Alginate hydrogels, *Dysidea robusta*, Marine bioactive compounds, Controlled release, Anti-inflammatory activity

## Abstract

Articular inflammatory processes are common in rheumatoid arthritis, osteoarthritis, and psoriatic arthritis. Standard therapy relies on oral anti-inflammatory drugs, which can cause systemic side effects with prolonged use. This has driven the search for natural alternatives and safer drug delivery systems. Intra-articular hydrogels enriched with bioactive compounds represent a promising approach, providing localized release of therapeutic molecules and reducing adverse effects. This study developed and characterized alginate (ALG)-based hydrogels enriched with bioactive compounds from the marine sponge *Dysidea robusta* and evaluated their anti-inflammatory potential in vitro. Hydrogels were prepared by dissolving ALG (1.5% and 3% w/v) and cross-linking with CaCO₃. Characterization included mass stability, scanning electron microscopy (SEM), and oscillatory rheology. Biological assays on fibroblasts and chondrocytes measured cytokine expression (IL-6 and TNF-α) by enzyme-linked immunosorbent assay (ELISA). The hydrogels showed initial mass gain followed by controlled degradation and a lamellar porous structure. Rheological analysis revealed tunable gelation and elasticity, while in vitro assays confirmed biocompatibility and a significant reduction of inflammatory markers. These findings indicate that ALG-based hydrogels containing *D. robusta* bioactives combine desirable physicochemical properties with anti-inflammatory activity, making them promising candidates for targeted drug delivery and tissue engineering.

**Supplementary Information:**

The online version contains supplementary material available at 10.1007/s12010-026-05684-z.

## Introduction

Articular inflammatory processes, commonly observed in conditions such as rheumatoid arthritis (RA), osteoarthritis (OA), and psoriatic arthritis, involve complex immune-mediated mechanisms that contribute to joint degeneration and functional impairment. These processes are characterized by synovial membrane hyperplasia, infiltration of inflammatory cells, and overproduction of pro-inflammatory cytokines, including tumor necrosis factor-alpha (TNF-α), interleukin-1β (IL-1β), and interleukin-6 (IL-6), which perpetuate local inflammation and cartilage degradation [1, 2].

The management of articular inflammatory processes has evolved significantly over recent decades, driven by advances in nonsteroidal anti-inflammatory drugs (NSAIDs) and glucocorticoids. Although the effectiveness in controlling acute inflammation, continued use of these drugs can lead to the occurrence of therapy-limiting side effects like gastrointestinal distress, renal toxicity, and even drug resistance [3].

In this context, discovering alternative means for delivering anti-inflammatory compounds is on high demand [4]. Anti-inflammatory metabolites from marine sponges, including terpenoids, alkaloids, peptides, and polyketides, have been studied using in vitro and in vivo models [5–7]. Around 84 anti-inflammatory substances obtained from marine sponges have been reported Magri et al. (2023) [7]. Terpenoids, alkaloids, peptides and polyketides are some of the major constituents isolated from marine sponges [7–9]. Mayer, Aviles, Rodriguez [10] demonstrated that five amphilectane metabolites [11–15] and two semi-synthetic derivatives [4, 16] from the marine sponge Hymeniacidon sp. (family Halichondriidae) displayed anti-inflammatory properties by inhibiting rat brain microglia thromboxane B2 (TXB2) synthesis via the cyclooxygenase-dependent mechanism.

Another therapeutical intervention that has gained attention for treating articular inflammation is the development of drug delivery systems based on polymeric matrices, especially using natural matrices and optimizing drug administration [17, 18]. Thus, alginate hydrogels have been emerging as an alternative for a more efficient system for delivering the anti-inflammatory compounds obtained from the sponges. Alginate hydrogels are three-dimensional cross-linked networks of hydrophilic polymers used for many biomedical applications, including drug delivery and tissue engineering. They are groups of linear anionic polysaccharides derived from kelp or *Sargassum* algae of brown algae and several bacterial strains [19]. Alginate hydrogels can carry bioactive compounds and have become the most widely used functional material for cell encapsulation and drug carriers. However, the ideal morphological and mechanical characteristics of alginate hydrogels for delivering bioactive metabolites from marine sponges are still to be investigated and explored. For example, Bonifacio et al. [20] have investigated the anti-inflammatory potential of hydrogels enriched with biocompounds of marine sponges (*Dysidea robusta*). The results showed that the hydrogels were stable and biocompatible and able of releasing the bioactive over time. Notably, the hydrogel enriched with the biocompound significantly reduced IL-6 levels, demonstrating an anti-inflammatory potential.

Thus, it is essential to the development of more efficient systems to deliver alternative anti-inflammatory molecules. For this, the hypothesis of the present study is that alginate-based hydrogel reticulated with calcium carbonate has compatible characteristics for delivering bioactive compounds obtained from *D. robusta* marine sponge. The aim of this study was to develop different alginate-based hydrogels and perform their characterization. Moreover, the in vitro biological activity of the alginate hydrogel enriched with bioactive compounds obtained from the marine sponge *D. robusta* was evaluated.

## Materials and Methods

### Hydrogel Preparation

To prepare the hydrogels, sodium ALG (MW 93 kDa) was dispersed in ultrapure water under magnetic stirring at 65 °C until complete dissolution, yielding ALG solutions at concentrations of 1.5% and 3.0% (w/v). Subsequently, CaCO₃ (99.5%, 0.6–0.7 μm) was added to the ALG solutions as the cross-linking agent, using the same CaCO₃ mass for all formulations, resulting in an ALG: CaCO₃ ratio based on mass (w/w). To promote CaCO₃ solubilization and initiate internal gelation, a freshly prepared glucono-δ-lactone (GDL) solution (1.5% w/v) was added in different volumes, as detailed in Table 1, to obtain ALG/CaCO₃:GDL volumetric ratios of 4:1, 5:1, and 6:1. The mixtures were homogenized for 20 s using magnetic stirring at room temperature. Subsequently, 500 µL of the pre-gelation ALG solution were transferred into molds and incubated in an oven at 37 °C for 1 h to allow complete gelation. The samples were then removed from the molds and subjected to further analyses.

**Table 1:** Manufactured hydrogel groups and their respective concentrations of ALG and CaCO_3_, along with the corresponding ALG: GDL ratio

Groups	ALG and CaCO_3_ (%)	ALG: GDL ratio
H1	1.5	4:1
H2	1.5	5:1
H3	1.5	6:1
H4	3.0	4:1
H5	3.0	5:1
H6	3.0	6:1

### Characterization of Hydrogel

#### Mass Stability

The mass stability was expressed as a relative mass variation index calculated according to Equation (Wf - Wi) / Wf × 100, where Wi corresponds to the initial mass and Wf to the mass at each time point. Samples were weighed on a precision scale, and the Wi was obtained. Samples were placed into tubes containing 10 mL of PBS (pH 7.4) and incubated for 1, 3, 6, 12, and 30 days in an incubator at 37 °C. After each experimental period, samples were removed from the tubes, dried in an incubator for 30 min and weighed to obtain the final mass (Wf) [17].

#### Scanning Electron Microscopy (SEM)

SEM was used for analyzing the hydrogel morphology (LEO 440, Carl Zeiss, Jena, Germany). The samples were observed at 15 kV accelerating voltage after being frozen with liquid nitrogen, lyophilized at − 30 °C, mounted on an aluminum base with carbon tape, and then gold-coated using a Cressington 108 Auto (Cressington, Watford, UK). The pore size was analyzed using the software ImageJ.

#### Rheological Analysis

The rheological properties of the hydrogels were evaluated using a Physica MCR 101 rheometer (Anton Paar, Austria). For the measurement of angular frequency sweep, the samples were gelled in 6-well culture plates for 24 h. Subsequently, the resulting discs were placed between parallel plates (PP25). For the flow curve, measurements were taken 3 min after the addition of the GDL. The measurements covered a range of deformation rates from 0 to 300 rad/s and a stress range from 0 to 20 Pa.

#### Collection and Processing of Marine Sponge *Dysidea robusta* (*D. robusta*)

*D. robusta* (Kingdom: Animalia; Phylum: Porifera; Class: Demospongiae; Subclass: Heteroscleromorpha; Order: Poecilosclerida; Family: Dysideidae; Genus: *Dysidea*; Species: *Dysidea robusta*) were collected at Prainha, in Arraial do Cabo, Rio de Janeiro (-22.960072°, -42.018200°). Sample collection was conducted in loco under authorization from SISBio (Biodiversity Authorization and Information System, Brazil; permit no. 28917-1) and SISGEN (National System for the Management of Genetic Heritage and Associated Traditional Knowledge; registration no. AEAF480). The sponges were rinsed with seawater and stored in seawater-filled thermal containers for transportation to the laboratory.

**Fig. 1:**
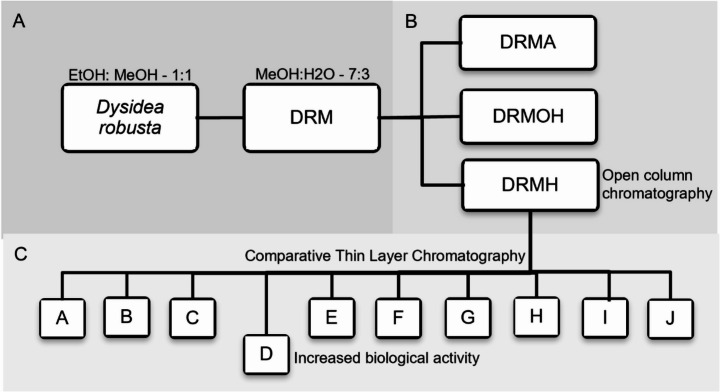
Diagram showing the steps involved in extracting bioactive substances from the marine sponge *D. robusta*, **A**: Obtaining the extract. **B**: Obtaining the partition phases. **C**: Fractionation of the partition phases

The marine sponge was macerated and extracted with a solution of ethanol and methanol (1:1) for 12 h. After filtration, the material was washed with methanol to obtain the methanolic extract. The extract (DRM) was resuspended in a hydroalcoholic solution (methanol: water − 7:3) and fractionated by liquid-liquid partitioning with hexane and ethyl acetate, giving rise to three fractions: hexane (DRMH), ethyl acetate (DRMA) and hydroalcoholic (DRMOH). The DRMH fraction, which showed the greatest biological activity, was selected for further fractionation using open column chromatography with Sephadex-LH 20 and gradient organic eluents. A total of 287 samples were obtained, grouped by chromatographic similarity into ten fractions (A to J). Among these, sub-fraction D stood out as having the greatest biological activity compared to the others and was selected for further analysis (Fig. 1). Sub-fraction D was analyzed by nuclear magnetic resonance (NMR) and mass spectrometry (MS) suggesting the presence of acylglycerol derivatives with predominance of 2-hydroxy-3-{[(8Z,11Z)-octacosa-8,11-dienoyl]oxy}propyl (8Z,11Z,14Z)-octacosa-8,11,14-trienoate, as previously reported [20].

#### Biocompound Incorporation into Hydrogel

For the in vitro test, two compositions of hydrogels (H3 and H6) were chosen based on the characterization data. Furthermore, the hydrogels were enriched with two different concentrations of Group D compounds from *D. robusta* (0.125 mg/mL and 0.25 mg/mL), as summarized in Table 2. The incorporation of these compounds into the ALG hydrogel involved physically mixing the compounds with the ALG and CaCO_3_ solution before adding GDL to ensure uniform distribution.

**Table 2:** Group D compounds incorporate into hydrogel

Groups	Gel composition	Concentration (mg/mL)	Incorporated material
H3	H3	0.0	-
H3DH	H3	0.250	Group D compounds
H3DL	H3	0.125	Group D compounds
H6	H6	0.0	-
H6DH	H6	0.250	Group D compounds
H6DL	H6	0.125	Group D compounds

### Bioactive Release Test

For the analysis of the controlled release of Group D compounds from the *D. robusta* methanolic extract, the UV–Vis absorption spectrum of the extract was initially analyzed, followed by the construction of a calibration curve in PBS using an Agilent Cary 60 UV–Vis spectrophotometer. For the controlled release assays, hydrogels synthesized as previously described were immersed in 50 mL of PBS (pH 7.4) and maintained at 37.5 °C. Aliquots of 500 µL were withdrawn at predetermined time points ranging from 15 min to 7 days, and the same volume was immediately replaced with fresh PBS to maintain a constant release medium volume and sink conditions. After each sampling, the absorbance of the collected aliquot was measured, and the cumulative amount of released compounds was calculated according to equation below.

$$\:{Q}_{t}=\:{C}_{t}V+{\sum\:}_{i=1}^{t-1}{C}_{i}{V}_{s}$$

Where $\:{Q}_{t}$is the cumulative amount of Group D compounds released at time *t*, $\:{C}_{t}$is the concentration measured at time *t*, $\:V$is the total volume of the release medium, $\:{C}_{i}$is the concentration measured at each previous sampling point, and $\:{V}_{s}$ is the volume of each withdrawn aliquot. The cumulative release was normalized to the total volume and expressed as the percentage of compound release. Release experiments were performed in duplicate.

Non-linear regression analysis (Korsmeyer-Peppas model).

The drug transport constants ($\:k$) and diffusion exponents (𝑛) of the different groups were determined by fitting the drug release test data to the Korsmeyer-Peppas equation [21]. M_t_ being the amount of drug released in time (t), $\:{M}_{{\infty\:}}$ was defined as the total theoretical amount of bioactive compound encapsulated in the hydrogel during formulation. This definition was adopted because none of the release profiles reached a plateau within the experimental timeframe. Even at the final time point (168 h), complete release of the encapsulated bioactive compound was not observed. The model fitting was performed using the cumulative release data obtained throughout the entire experimental period, considering 168 h as the total evaluated release time. Microsoft Office Excel (Microsoft Corporation, Redmond, USA) was used to calculate the values of k and n by linear regression of the logarithmic form of the Korsmeyer–Peppas equation.

$$\:{M}_{t}/{M}_{\infty\:}=k{t}^{\mathrm{n}}$$

### Cell Culture

Three cell types were utilized in this study: fibroblasts (L929), chondrocytes (primary lineage) and macrophages (RAW 264.7). Fibroblasts and macrophages were obtained from the Rio de Janeiro Cell Bank (BCRJ, RJ, Brazil), while chondrocytes were extracted from the articular cartilage of Wistar rats. All cell lines were cultivated under standard conditions (37 °C in a humidified atmosphere with 5% CO_2_) in DMEM medium or RPMI supplemented with 10% fetal bovine serum (FBS), 100 U/mL penicillin, and 100 µg/mL streptomycin.

### Conditioned Medium Preparation

The hydrogel was prepared under controlled flow conditions using reagents previously sterilized by exposure to UV light for 24 h and subsequently integrated into Falcon tubes containing DMEM or RPMI culture medium supplemented with 10% fetal bovine serum. The hydrogel concentration was set at 5% (w/v), calculated based on the wet mass of the hydrogel. These preparations were incubated in a CO₂ incubator at 37 °C. After 24 h of incubation, the conditioned medium was filtered through a 0.22 μm pore-size membrane (Kasvi, Brazil) to ensure sterility and remove any particulate matter.

### Metabolic Activity

Cell viability was assessed by the metabolic activity of the cells using the AlamarBlue assay. Cells (L929 and chondrocyte) were seeded in 48-well plates at a density of 1 × 10^4^ cells per well. After cell adhesion, the DMEM medium was replaced with conditioned medium. Cells were maintained for different experimental periods (1, 3, and 6 days) in a CO_2_-controlled incubator at 37 °C. After each incubation period, 10% AlamarBlue solution was added to each well, and incubated for 2 h 30 min. Then, 200 µL of the solution was transferred to 96-well plates for absorbance readings in the 570–600 nm range, using a microplate spectrophotometer (Bio-Tek Instruments, USA).

#### ELISA

The anti-inflammatory potential of the hydrogel enriched with the compounds from *D. robusta* was evaluated using the ELISA test with RAW 264.7 macrophage cell cultures in a pro-inflammatory state at experimental time points of 1 and 3 days. For this purpose, the cells were treated with 2 µg/mL of Escherichia coli lipopolysaccharide (E. coli, LPS) (Sigma, USA) for 24 h to induce the M1 phenotype. The experimental groups included macrophages exposed to hydrogels loaded with the compounds (H3, H6, H3DH, H3DL, H6DH, and H6DL) and a Control group treated with the LPS alone.

The ELISA test was employed to assess the concentration of IL-6 and TNF-α. Culture medium samples were added to the wells of ELISA plates containing antibodies specific to IL-6 or TNF-α, followed by incubation for 2 h at room temperature. After washing, 100 µL of the secondary antibody was added to each well, and the plate was incubated for another 2 h. Following a second wash, 100 µL of treated TMB was added to each well for a third incubation of 15 min in the dark. The reaction was stopped with sulfuric acid, and the absorbance was measured at 450 nm.

### Statistical Analysis

Statistical analyses were performed using GraphPad Prism software (GraphPad Software Inc., San Diego, USA). Data are presented as mean ± standard deviation. Comparisons among experimental groups were performed using two-way analysis of variance (two-way ANOVA), considering treatment and time as independent factors, followed by Tukey’s multiple comparisons test. Differences were considered statistically significant when *p* < 0.05. Regarding statistical power, no formal a priori power analysis was performed. The number of replicates was defined based on previous studies in the literature that used similar experimental designs. To clarify the number of replicates performed to the reader, we added this description in the statistical analysis.

## Results

### Mass Stability

The stability of hydrogels was assessed through changes in mass over time (Table 3). On the first day, H1 exhibited significantly higher mass loss compared to H2 (*p* = 0.0322) and H4 (*p* = 0.0096), but less than H3 (*p* = 0.0190). H2 had higher mass loss than H3 (*p* = 0.0190) but less than H4 (*p* = 0.0322), while H3 showed lower mass loss compared to H4 (*p* = 0.0088). Additionally, H4 had significantly higher mass loss compared to H6 (*p* = 0.0096). By the third day, H1 showed greater mass loss than H3 (*p* = 0.0036), H5 (*p* = 0.0004), and H6 (*p* = 0.0005). H2 also exhibited greater mass loss compared to H3 (*p* = 0.0106), H5 (*p* = 0.0002), and H6 (*p* = 0.0053). H3 had lower mass loss compared to H5 (*p* = 0.0014) and H6 (*p* = 0.0375). On the sixth day, H2’s mass loss was greater than that of H4 (*p* = 0.0014), H5 (*p* = 0.0085), and H6 (*p* = 0.0044). Similarly, H3 exhibited greater mass loss compared to H4 (*p* = 0.0192), H5 (*p* = 0.0223), and H6 (*p* = 0.0117). On the twelfth day, H2 and H3 both showed significantly higher mass loss compared to H4 (*p* = 0.0044 and *p* < 0.001, respectively). By the thirtieth day, H2 had significantly higher mass loss than H4 (*p* < 0.001), while no significant differences were found among the remaining groups.

**Table 3:** The percentage changes in mass of 1.5% and 3% ALG hydrogels at different time points. Statistical differences represented by: (*) compared to H1; (&) compared to H2; (#) compared to H3 and (%) compared to H4

Days	ALG 1.5%			ALG 3%		
	H1	H2	H3	H4	H5	H6
1	27.43 ± 3.11	48.1 ± 7.12*	21.14 ± 1.53*^&^	81.35 ± 11.83*^&#^	85.94 ± 13.84	36.78 ± 6.99^%^
3	46.22 ± 2.31	53.12 ± 3.73	70.79 ± 4.85	75.36 ± 13.58	103.04 ± 5.9*^&#^	94.29 ± 8.67*^&#^
6	12.63 ± 5.72	12.81 ± 3.49	32.32 ± 7.78	58.6 ± 7.23*^&#^	70.09 ± 12.31*^&#^	69.76 ± 10.52*^&#^
12	41.16 ± 13.85	47.55 ± 5.15	36.72 ± 1.27	73.7 ± 2.1^&#^	60.44 ± 15.28	50.77 ± 10.83
30	-35.15 ± 5.05	-42.57 ± 2.08	-27.61 ± 6.11	-25.95 ± 4.87^&^	-42.52 ± 24.11	-19.04 ± 8.89

### SEM

**Fig. 2:**
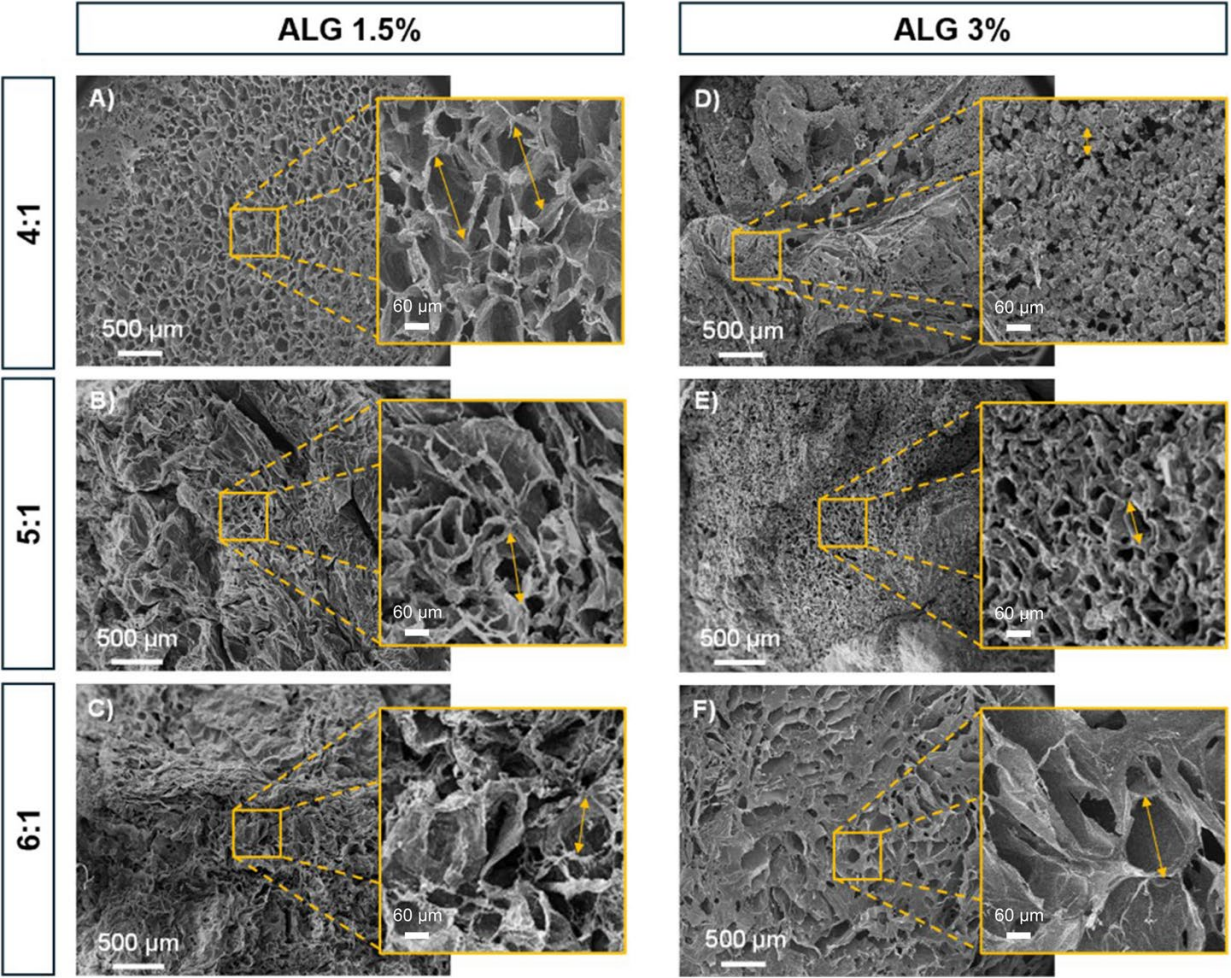
Microstructures of lyophilized ALG hydrogels observed using SEM. (**A**) H1, (**B**) H2, (**C**) H3, (**D**) H4, (**E**) H5, and (**F**) H6. Yellow arrows indicate porosity on the surface of the hydrogels

SEM analyses of the lyophilized samples were conducted (Fig. 2). All gels exhibit lamellar structures that interconnect to form micropores. The observed pore sizes were 238 ± 128 μm, 186 ± 92 μm, 128 ± 58 μm, 72 ± 44 μm, 111 ± 37 μm, and 219 ± 32 μm for gels H1, H2, H3, H4, H5, and H6, respectively. At lower concentration of Ca^2+^ cross-linker the higher amounts of GDL leads to higher pore sizes between the gel lamellae. At higher concentration of Ca^2+^ cross-linker the higher amounts of GDL leads to lower pore sizes between the gel lamellae.

Oscillatory rheology.

### Time Sweep

**Fig. 3:**
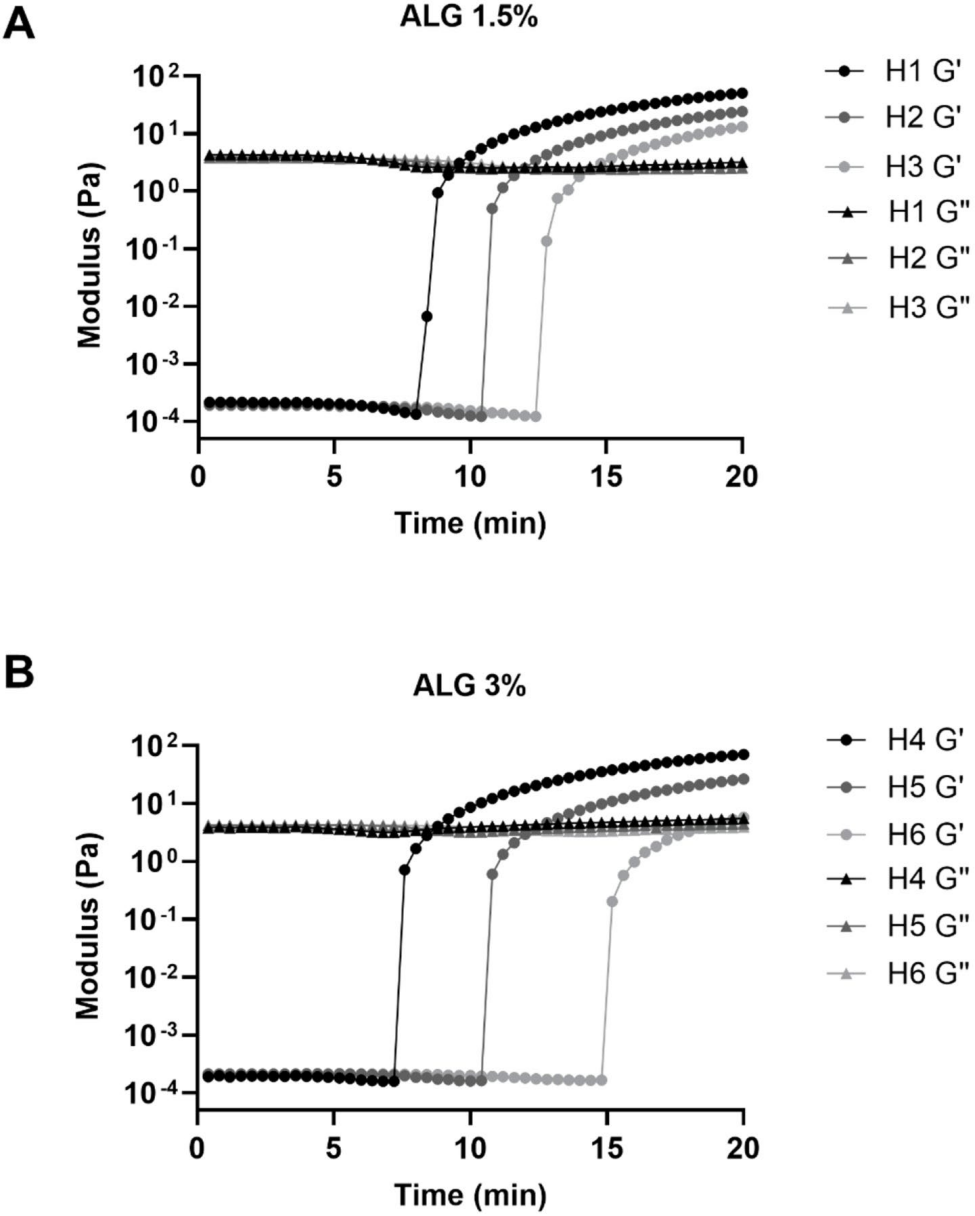
Dynamic changes in viscoelastic properties of 1.5% (**A**) and 3% (**B**) ALG hydrogels over time

Figure 3 shows the time sweep curves of the hydrogels, illustrating the evolution of the storage (G’) and loss (G’’) moduli during gelation. For hydrogels synthesized with 1.5% Ca^2+^ cross-linker (Fig. 3A), the intersection of G’ and G’’, marking the transition from a predominantly liquid-like to a solid-like structure, occurred at 9.6 min for H1, 12.4 min for H2, and 14.8 min for H3. In contrast, for hydrogels with 3% Ca^2+^ cross-linker (Fig. 3B), the crossover points were observed at 8.8 min for H4, 12.4 min for H5, and 18.8 min for H6.

### Frequency Sweep

**Fig. 4:**
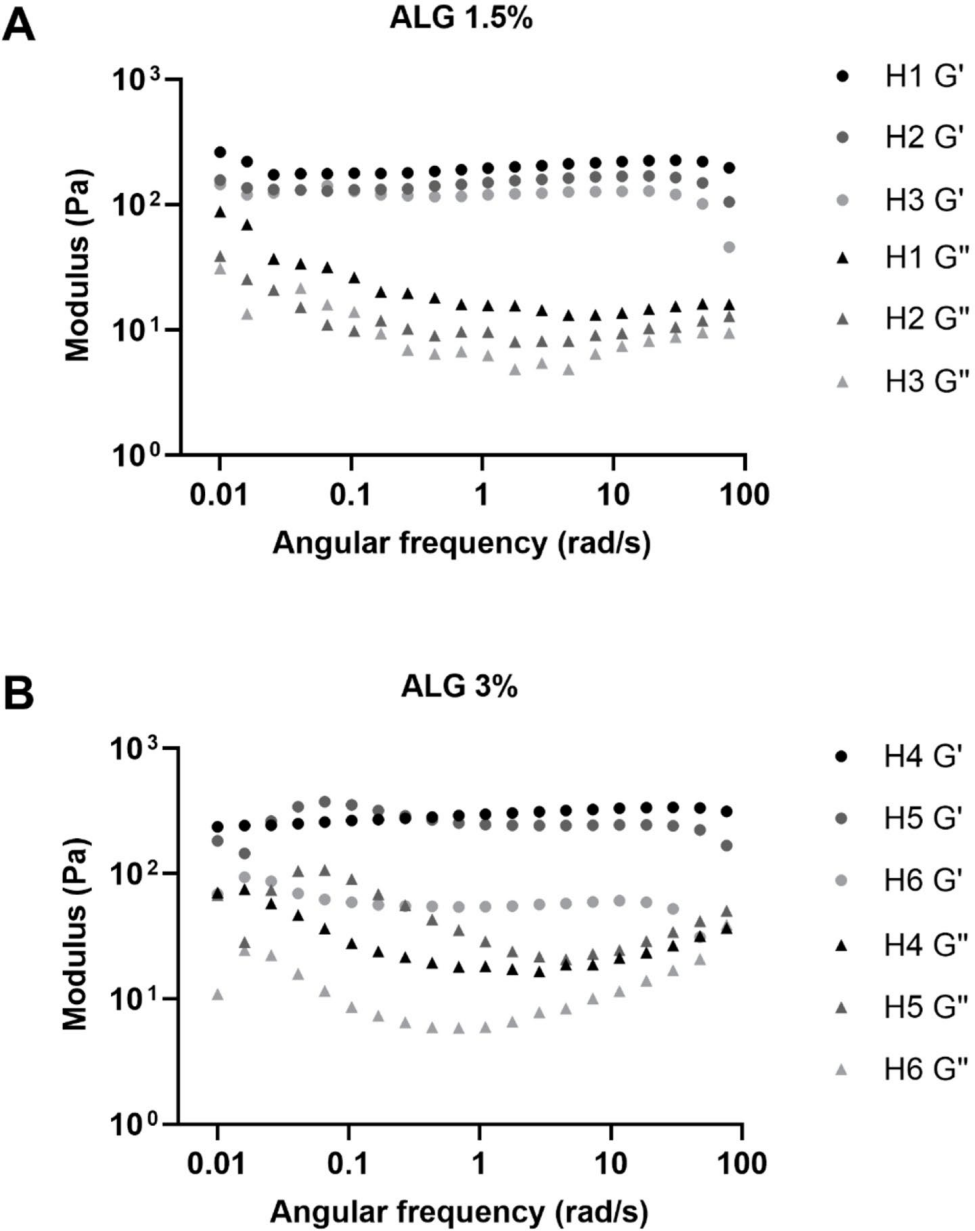
G′ and G″ of 1.5% (**A**) and 3% (**B**) ALG hydrogels by angular frequency sweep from 0.01 to 100 rad/s

Figure 4 presents the frequency sweep profiles of the hydrogels, showing their viscoelastic behavior over a range of angular frequencies. Testing various formulations indicated that hydrogels with higher ALG-to-GDL ratios (5:1 and 6:1) and 1.5% Ca^2+^ maintained a robust elastic structure, as reflected in the consistent G’ exceeding 100 Pa, with low viscosity G’’ at high frequencies (Fig. 4A). In contrast, increasing the Ca^2+^ concentration to 3% resulted in greater mechanical complexity, especially with 4:1 and 5:1 ratio, where the loss G’’ exhibited an increase at low frequencies, indicating a more pronounced viscoelastic response (Fig. 4B). Interestingly, the formulation with the highest ALG-GDL ratio (6:1) and 3% Ca^2+^ showed a significant reduction in elastic rigidity, suggesting saturation in cross-linking capacity or a less efficient gel structure.

### Bioactive Release Test

**Fig. 5:**
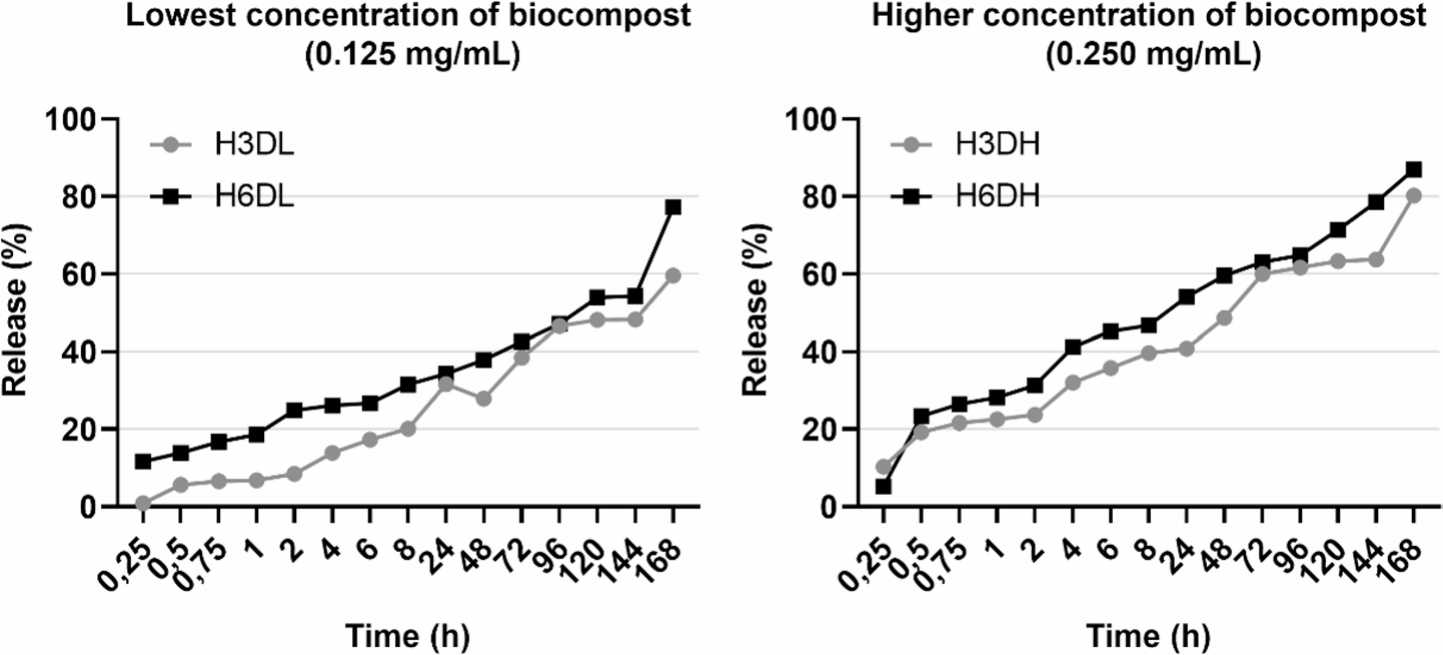
Release profiles of Group D compounds from *D. robusta* of ALG hydrogels expressed as percentage wt% of released actives as a function of time

In Figure S1A, it can be observed that Group D compounds from *D. robusta* absorbs in the UV region, with a maximum absorption at 207 nm. Based on this result, a calibration curve was constructed by a 10-fold serial dilution in triplicate, starting from the maximum volume of the compounds added to the hydrogel (Figure S1B). The equation of the obtained curve was y = 0.87698x + 0.16747, with an R^2^ of 0.9942, indicating a good linear fit. In Fig. 5, the release profiles for the samples loaded with Group D compounds from *D. robusta* at two different concentrations can be observed. It is important to highlight that the H3DL and H6DL samples contain half the concentration of Group D compounds from *D. robusta*. For a better interpretation of the release graphs, their maximum release is 50% compared to the H3DH and H6DH samples (100%). For both groups with varying concentrations, a rapid release is observed during the first 8 h, with 39%, 10%, 47%, and 16% release for the H3DH, H3DL, H6DH, and H6DL samples, respectively. After this period, a slow, gradual release occurs for the H3DL and H6DL groups, which contain the lower concentration of Group D compounds from *D. robusta*, reaching maximum releases of 30% and 39%, respectively. For the H3DH and H6DH samples, the release continues at a more pronounced rate over the days, reaching 80% and 87% of the Group D compounds from *D. robusta* by the end of the 7th day, respectively.

### Non-linear Data Fitting Using the Korsmeyer-Peppas Model

From the data fit, the transport constants and transport exponents were determined (see Supplementary Information, Figure S2) using the Korsmeyer-Peppas model, that is an empirical model widely used to describe drug release from polymeric matrix systems. The H6DH group showed the highest *k* value (0.2884) and an *n* of 0.1999, with an R^2^ of 0.97, indicating a rapid release and a highly adjusted model. The H3DH group showed *k* = 0.2065, *n* = 0.2457 and R^2^ = 0.94. The H6DL group showed *k* = 0.0891, *n* = 0.2338 and R^2^ = 0.95, while the H3DL group showed the lowest values of *k* (0.0380) and R*2* (0.88), with *n* = 0.3947. The fit of the model was considered satisfactory for all groups, with R^2^ values between 0.88 and 0.97 (Table 4).

**Table 4:** Modeling the release kinetics of bioactive compounds extracted from the marine sponge *D. robusta* and incorporated into alginate hydrogels, using the Korsmeyer-Peppas equation

Groups	Drug transport constants ($\:\boldsymbol{k}$)	Diffusion exponents (𝑛)	*R* ^2^
H3DH	0,206538	0,2457	0,94
H3DL	0,038019	0,3947	0,98
H6DH	0,288403	0,1999	0,97
H6DL	0,089125	0,2338	0,95

### Metabolic Activity of Fibroblasts

**Fig. 6:**
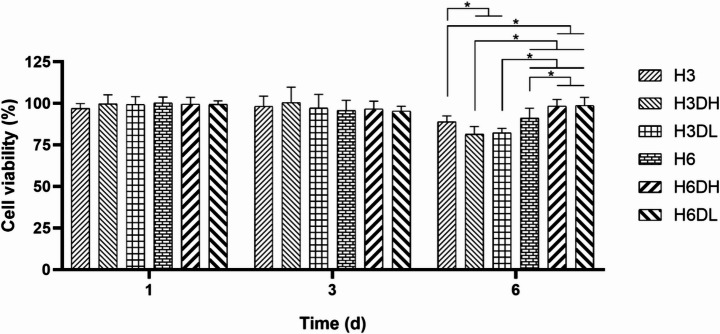
Metabolic activity of fibroblasts exposed to Group D compounds from D. robusta at different time points. Data are expressed as mean ± standard deviation. Statistically significant differences compared to the control group were observed (*p* < 0.05)

Figure 6 illustrates the outcomes of fibroblast metabolic activity when cultured in indirect contact with hydrogel samples enriched with Group D compounds from *D. robusta*. The values are expressed as percentages relative to the control, which was set at 100%. It is possible to observe that no statistically significant differences were observed among any groups at the experimental periods of one and three days (*p* ≥ 0.05). However, on 6th day, H3 displayed increased metabolic activity compared to H3DH and H3DL and lower values compared to GE and H6DL. Both H3DH and H3DL exhibited lower metabolic activity compared to H6, H6DH, and H6DL. H6 showed lower metabolic activity compared to both H6DH and H6DL.

### Metabolic Activity of Chondrocytes

**Fig. 7:**
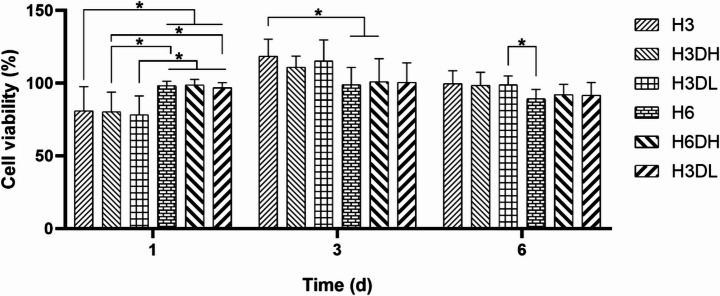
Metabolic activity of chondrocytes exposed to Group D compounds from D. robusta over time. Results are presented as mean ± standard deviation. Statistically significant differences in relation to the control group are indicated (*p* < 0.05)

Figure 7 presents the results of chondrocyte metabolic activity in cultures exposed indirectly to hydrogel samples enriched with Group D compounds from *D. robusta*. Similarly to the fibroblast viability assay, all values are expressed as percentages relative to the control, which was set at 100%. In the first experimental period, H3 exhibited lower values than H6 and H6DH. Additionally, H3DH displayed reduced activity compared to H6, H6DH, and H6DL, whereas H3DL showed the lowest activity compared to H6, H6DH, and H6DL. After 3 days, H3 had higher activity than both H6 and H6DL. By the sixth day, a significant difference was only found between H3DL and H6, with no further significant changes between other groups.

#### ELISA

**Fig. 8:**
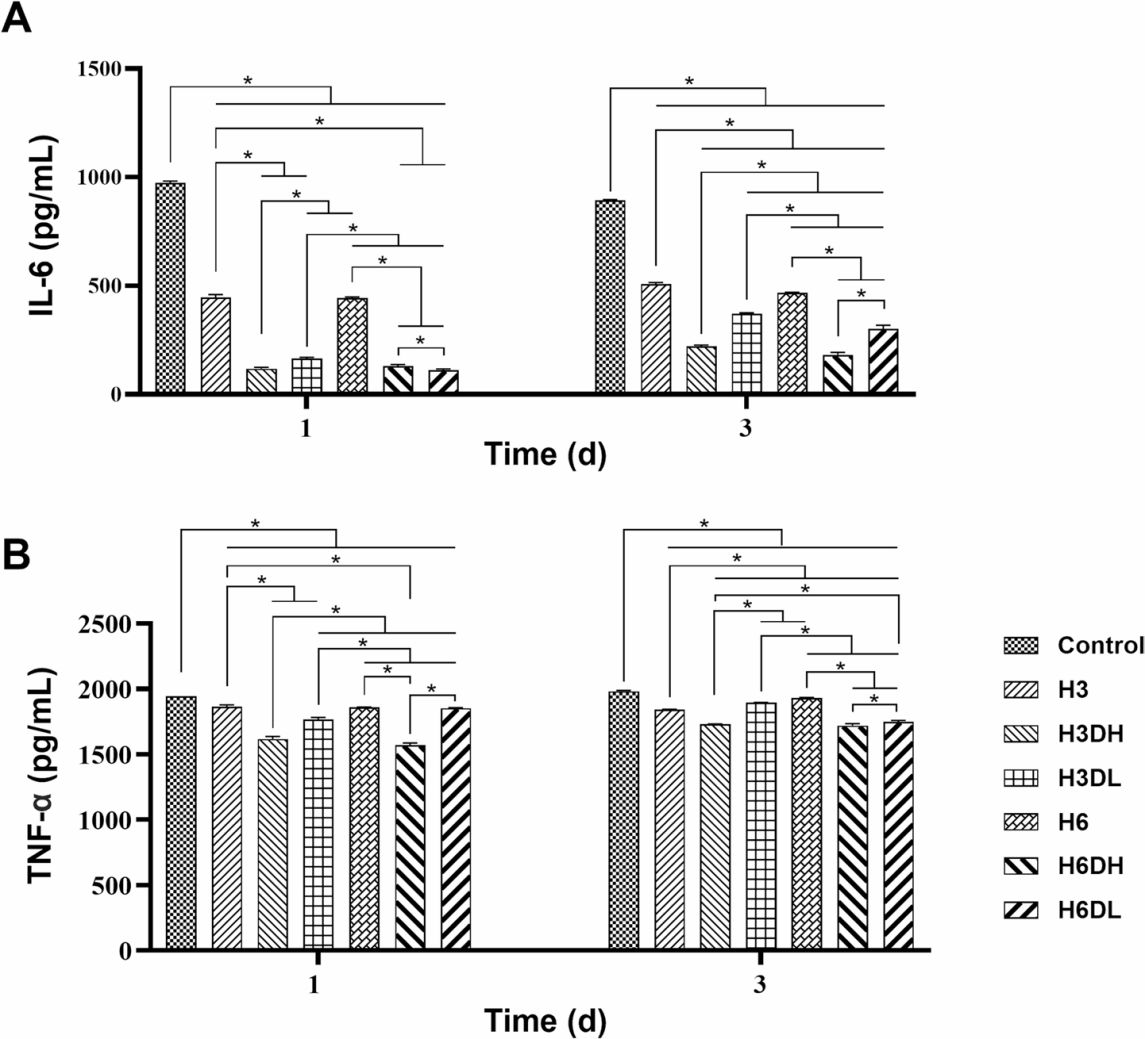
ELISA quantification of cytokine levels in macrophage cultures subjected to different experimental conditions. (**A**) IL-6 and (**B**) TNF-α levels. Data are presented as mean ± standard deviation. Statistically significant differences between groups are indicated (*p* < 0.05)

Figure 8 shows the levels of pro-inflammatory cytokines over time, with Fig. 8A presenting IL-6 levels. On day 1, the Control group exhibited the highest IL-6 levels. H3 showed higher values than H3DH, H3DL, H6DH, and H6DL. H3DH presented lower levels than H3DL and H6, while H3DL was higher than H6 but lower than H6DH and H6DL. H6 exhibited higher levels than H6DH and H6DL, with H6DH remaining lower than H6DL. By day 3, the Control group continued to show the highest IL-6 levels. H3 remained higher than all other groups, while H3DH showed the lowest levels compared to H3DL, H6, H6DH, and H6DL. H3DL remained lower than H6 but higher than H6DH and H6DL, and H6 continued to present higher levels than H6DH and H6DL.

Figure 8B depicts TNF-α levels, showing a pattern similar to IL-6. On day 1, the Control group exhibited the highest levels. H3 presented higher TNF-α levels than H3DH, H3DL, and H6DH. H3DH showed lower levels than H3DL, H6, and H6DL, but higher than H6DH. H3DL remained lower than H6 and H6DL, yet higher than H6DH. H6 showed higher levels than H6DH and H6DL, with H6DH remaining lower than H6DL. By day 3, the Control group continued to present the highest TNF-α levels. H3 remained higher than H3DH, H6DH, and H6DL, but lower than H3DL and H6. H3DH stayed lower than H3DL and H6, while remaining higher than H6DH and H6DL. H3DL continued to be lower than H6 but higher than H6DH and H6DL, and H6 maintained higher levels than H6DH and H6DL.

## Discussion

The present study evaluated the physical and morphological characteristics of different compositions of hydrogels of ALG to develop an optimized drug delivery system with bioactive compounds obtained from the marine sponge *D. robusta* with anti-inflammatory effects. The results of mass stability demonstrated that hydrogels exhibited a gain of mass followed by mass loss. Moreover, pH measurement showed a decrease in the first periods of evaluation followed by stabilization. Rheology showed that there were dynamic changes in viscoelastic properties, with the transition from liquid to solid structure between 9.6 and 14.8 min. The higher proportions of alginate to GDL (5:1 and 6:1) maintained a robust elastic structure. In addition, all the gel compositions analyzed started releasing the biocompounds within the first 15 min and continued releasing it gradually for up to 7 days. For fibroblast metabolic activity, similar results for all groups were seen in the first experimental periods and increased values were found for H3. Moreover, H3DH and H3DL exhibited lower metabolic activity. For chondrocyte metabolic higher values were found for groups H6 compared to others, especially the first day of experiment. IL-6 and TNF-α levels demonstrated lower values in H6DH and H6DL compared to the other groups.

The results demonstrated that hydrogels exhibited a gain in mass in the beginning of the experiments followed by a mass loss. It can be suggested that the use of Ca^2+^ cross-linker ate the concentration of 3% enhances water retention, as evidenced by the higher weight in comparison to the group with a lower Ca^2+^ cross-linker rate (1.5%). However, from 6th day onwards, a destabilization of the three-dimensional network of the gels occurs, possibly due to the migration of Ca^2+^ into the medium, leading to mass loss and, consequently, a reduction in the gel ability to retain water molecules. S. Liu et al. (2016) [22] observed that the greater stability of Nd^3+^-crosslinked alginate hydrogels was due to the reduced leaching of the cross-linker compared to those crosslinked with Ca^2+^ [22]. These results align with those of the present study, demonstrating the destabilization of the alginate structure due to the leaching of Ca^2+^ over time.

Modifications in the gel formulation can significantly alter their microstructure and, consequently, their properties. SEM analyses reveal that the microstructure of the gels was influenced by both Ca^2+^ and GDL. Probably, in the alginate egg-box model an increased number of cross-linkers creates more junction points between alginate monomers [23]. Changes in the amount of GDL also impact the microstructure of the lyophilized gels, as GDL acts as a gelling agent [24]. With less available Ca^2+^, gel formation is slower, allowing more gradual control over the gelation process, resulting in a more uniform gel [25]. Conversely, a higher amount of Ca^2+^ accelerates gelation since more calcium ions are available to interact with the alginate, potentially leading to faster but less uniform gelation.

Rheology is a technique used for evaluating the flow and deformation behavior of gels. The results from time sweep tests show that for these formulations, it was observed that a low dependence on the Ca^2+^ cross-linker concentration but a high dependence on the amount of GDL. As expected, lower amounts of GDL prolong the time required for gel formation, a trend reflected in the later crossover of G’ and G’’ [26]. The frequency sweep results reveal that the stability and mechanical properties of alginate hydrogels are significantly influenced by the alginate to GDL ratio and the Ca^2+^ ion concentration [27, 28]. The findings of this analysis suggest that increasing the alginate-GDL ratio from 4:1 to 5:1 with 1.5% Ca^2+^ is optimal for producing hydrogels with high elasticity and stability, while a higher Ca^2+^ concentration, despite increasing viscoelastic complexity, requires careful adjustment of the alginate ratio to avoid less dense gel networks and reduced rigidity. Modulating these parameters allows for optimizing the mechanical properties of alginate hydrogels. Especially when incorporating bioactive agents, a slower gel formation with reduced elastic rigidity could be advantageous to achieve a more uniform distribution of the agent throughout the gel three-dimensional network, facilitating handling for potential applications with syringes.

In addition, hydrogels with a higher amount of the Group D biocompounds from *D. robusta* exhibit greater release compared to those that received reduced amounts, even after normalization. Among the groups with 1.5% and 3% Ca^2+^ as the crosslinking agent, the hydrogels with the higher percentage of crosslinkers tend to sustain the release over time and achieve higher release values compared to those with lower amounts. This behavior can be attributed to the physicochemical properties of hydrogels, particularly their rheological characteristics. The gels with 1.5% crosslinking agent form a less rigid three-dimensional network than those with 3%, as the higher amount of Ca^2+^ increases the crosslinking density. Consequently, the 1.5% Ca^2+^ samples have more active sites available for crosslinking compared to the 3% samples. Group D compounds from *D. robusta* can interact with the polymer chains of the hydrogel, and since the crosslinking density of the 1.5% Ca^2+^ sample is lower, a larger portion of the Group D compounds from *D. robusta* may remain trapped within the hydrogel structure. Regarding the release profile of the compounds, a rapid release is observed in the first 8 h, followed by sustained release over several days, particularly for the gels with a higher amount of the Group D compounds from *D. robusta*. This profile may have promising implications for clinical applications, as the initial rapid release can enhance the active ingredient’s penetration, while the sustained release ensures a prolonged therapeutic effect.

Modeling the profile release kinetics of bioactive compounds extracted from the marine sponge *D. robusta* and incorporated into alginate hydrogels, using the Korsmeyer-Peppas equation, revealed significant differences between the groups evaluated. The parameter *k*, which represents the drug’s transport constant, was higher in the H6DH group, indicating a higher release rate. In contrast, the H3DL group had the lowest *k* value, suggesting a slower release of the active ingredient. This trend shows that the concentration of the biocompounds directly influences the release rate, with higher concentrations promoting higher *k* values, especially in less dense matrices. The concentration of alginate, on the other hand, acts as a physical barrier, slowing down diffusion, which is evidenced by the lower *k* value in the H6DL group compared to H3DH. As for the diffusion exponent *n*, used to identify the release mechanism, all the values were below 0.5, indicating Fickian-type transport (controlled diffusion), according to the classic criteria in the literature [29]. The lowest values were observed in the H6DH and H6DL groups, suggesting that the increase in alginate concentration resulted in an even more controlled system by simple diffusion. The H3DL group showed the highest value of n, close to the limit between Fickian diffusion and anomalous transport, which may indicate a slightly greater contribution of matrix relaxation to the release of the compound. Taken together, these results suggest that the release rate can be modulated by manipulating the proportions of alginate and incorporated biocompounds, allowing the system to be adjusted according to therapeutic needs. Hydrogels with a higher biocompounds content, especially at lower alginate concentrations, promote faster release, while high alginate concentrations tend to restrict drug diffusion, favoring prolonged release [30, 31].

Anti-inflammatory metabolites from marine sponges have been studied by many researchers [7, 32]. In a systematic review, Magri et al. (2023) [7], have stated that metabolites from marine sponges present anti-inflammatory activity and biocompatibility, especially terpenoids, alkaloids, peptides, and polyketides. In the present study, the in vitro studies demonstrated that, for both cell lineages, all the hydrogels tested with Group D compounds from *D. robusta* can be considered non-cytotoxicity (values found for this variable were above 70% for both treated groups compared to control group in most experimental conditions). Following the ISO 109333-5:2009, a material to be used for medical proposals can be considered toxic for cells if it produces more than 30% of death. In this context, the in vitro data of the present study highlights the biocompatibility of the system hydrogels loaded with the bioactive metabolites in both cell lineages evaluated.

In addition, cytokines such as IL-6, IL-1β, TNF-α, and transforming growth factor-β are produced by macrophages and monocytes during inflammatory processes [33]. Among them, IL-6 and TNF-α play a central role in acute inflammation and contribute to the timely resolution of wound healing [34], being involved in the regulation of pro-inflammatory responses in several cell types, including macrophages, stromal cells, and endothelial cells, and in the transition to a reparative environment [35]. In the present study, the ELISA assay showed that the negative control group presented the highest levels of both IL-6 and TNF-α, whereas hydrogels loaded with the higher concentration of compounds showed the lowest values, indicating an anti-inflammatory effect of the bioactive metabolites from *D. robusta*. This effect was more pronounced for IL-6, while TNF-α remained relatively elevated throughout the experimental period, suggesting a less marked modulation of this cytokine. This difference may be related to the more complex regulation of TNF-α during inflammatory responses and to the distinct signaling pathways involved in the control of each cytokine, since the compounds may affect JAK/STAT-related signaling more strongly than NF-κB-mediated pathways [36]. Similar findings have been reported for other marine-derived compounds. For example, Hu et al. [37] demonstrated that dysiarenone, isolated from *Dysidea arenaria*, suppressed the production of IL-6 and TNF-α and inhibited the phosphorylation of MAPKs, including p38 and ERK, as well as Akt and NF-κB p65. Likewise, cyanogramide, isolated from a marine actinomycete, inhibited IL-6 production in LPS-stimulated RAW264.7 cells, reinforcing the therapeutic potential of marine compounds in inflammatory modulation [38]. Seo et al. [39] also demonstrated reduced LPS-induced production of inflammatory cytokines and inhibition of NF-κB transcriptional activity in the presence of phorbaketal A, isolated from the marine sponge *Phorbas* sp. In agreement with the present findings, Chaudhari et al. (2020) [36]. reported that sponge-derived compounds markedly reduced IL-6 levels in RAW 264.7 macrophages, while their effect on TNF-α was less evident. Together, these findings reinforce the anti-inflammatory potential of compounds obtained from marine sponges, including those from *D. robusta*, while also indicating that their effects may vary according to the cytokine evaluated.

With the need of developing drug delivery systems, the manufacturing of hydrogels able of releasing anti-inflammatory compounds for treating diseases are of high demand [10, 40]. In this context, the results indicate a clear relationship between hydrogel composition and biological performance. The higher polymer concentration used in the H6 formulations promoted an increase in crosslinking density, resulting in a more compact network with reduced pore size. This structural organization directly influenced the release profile of the encapsulated bioactive compounds, leading to a slower and more controlled release over time, particularly in the H6DH and H6DL formulations. The sustained release behaviour was reflected in in vitro assays, where these formulations exhibited lower levels of pro-inflammatory cytokines compared to less crosslinked hydrogels. Together, these findings demonstrate that the improved crosslinking and structural stability of the H6DH and H6DL hydrogels contributed to better control of bioactive release and a superior anti-inflammatory profile, reinforcing the importance of hydrogel composition in modulating physicochemical properties and biological outcomes.

These findings highlight the potential of this system as a suitable material to be used for tissue engineering applications. However, further in vivo studies are necessary using long-term evaluation to investigate the effects of the implantation of alginate hydrogels loaded with marine compounds in animal models.

## Conclusion

This study developed ALG-based hydrogels and enriched with bioactive compounds extracted from the marine sponge *D. robusta*, aiming to evaluate the efficacy of the hydrogel as delivery systems and the anti-inflammatory potential of the compounds. The H6DH and H6DL stood out for its stable physical and morphological properties essential for controlled release of bioactive compounds. In vitro assays confirmed the biocompatibility and anti-inflammatory action of the compounds, with emphasis on the reduction of IL-6 and TNF-α for both H6DH and H6DL This group also preserved the ideal viscoelastic properties of the hydrogel. Despite the promising results, additional in vivo studies are needed to validate the long-term efficacy and clinical applicability.

## Supplementary Information

Below is the link to the electronic supplementary material.

Supplementary Material 1

Supplementary Material 2

## Data Availability

The data supporting the findings of this study are available from the corresponding author upon reasonable request.

## Abbreviations

RA: rheumatoid arthritis
OA: osteoarthritis
TNF-α: tumor necrosis factor-alpha
IL-1β: interleukin-1β
IL-6: interleukin-6
NSAIDs: nonsteroidal anti-inflammatory drugs
TXB2: thromboxane B2
ALG: sodium alginate
CaCO_3_: calcium carbonate
MW: molecular weight
kDa: kilodalton
GDL: glucono-D-lactone
µL: microliter
sec: second
h: hour
PBS: phosphate-buffered saline
Wi: initial weight
Wf: final mass
SEM: Scanning Electron Microscope
kV: kilovolt
rad/s: radians per second
Pa: pascal
SISBio: Sistema de Autorização e Informação em Biodiversidade
SISGEN: Sistema Nacional de Gestão do Patrimônio Genético e do Conhecimento Tradicional Associado
DRM: extract
DRMH: hexane
DRMA: ethyl acetate
DRMOH: hydroalcoholic
MeOH: methanol
UV-Vis: ultraviolet visible spectroscopy
°C: degree Celsius
min: minutes
k: transport constants
n: diffusion exponents
t: time
CO_2_: carbon dioxide
FBS: fetal bovine serum
U/mL: unit per milliliter
DMEM: Dulbecco’s modified Eagle’s medium
µm: micrometer
µg/mL: micrograms per milliliter
LPS: Escherichia coli lipopolysaccharide
nm: nanometer
Ca^2+^: calcium ions
ISO: International Organization for Standardization
MMPs: metalloproteinase
NF-κB: NFAT, AP-1,transcription factors

## References

[1] McInnes, I. B., & Schett, G. (2011). The Pathogenesis of Rheumatoid Arthritis. *New England Journal of Medicine*, *365*, 2205–2219. 10.1056/NEJMra1004965 22150039 10.1056/NEJMra1004965

[2] Firestein, G. S., & McInnes, I. B. (2017). Immunopathogenesis of Rheumatoid Arthritis. *Immunity*, *46*, 183–196. 10.1016/j.immuni.2017.02.006 28228278 10.1016/j.immuni.2017.02.006 PMC5385708

[3] Latourte, A., Kloppenburg, M., & Richette, P. (2020). Emerging pharmaceutical therapies for osteoarthritis. *Nature Reviews Rheumatology*, *16*, 673–688. 10.1038/s41584-020-00518-6 33122845 10.1038/s41584-020-00518-6

[4] Fürst, R., & Zündorf, I. (2014). Plant-derived anti-inflammatory compounds: hopes and disappointments regarding the translation of preclinical knowledge into clinical progress. *Mediators Inflamm*, *2014*, 146832. 10.1155/2014/146832 24987194 10.1155/2014/146832 PMC4060065

[5] Hwang, B. S., Lee, K., Yang, C., et al. (2013). Characterization and Anti-inflammatory Effects of Iodinated Acetylenic Acids Isolated from the Marine Sponges *Suberites mammilaris* and *Suberites japonicus*. *Journal Of Natural Products*, *76*, 2355–2359. 10.1021/np400793r 24256436 10.1021/np400793r

[6] A. J (2013). Impact of Quercetin, Diallyl Disulfide and Nimbolide on the Regulation of Nuclear Factor Kappa B Expression in Prostate and Breast Cancer Cell Lines. *Natural Products Chemistry & Research*, *1*. 10.4172/2329-6836.1000115

[7] Magri, A. M. P., Avanzi, I. R., Vila, G. T., et al. (2023). Anti-inflammatory Effects of Compounds Extracted from Marine Sponge s: A Systematic Review. *Antiinflamm Antiallergy Agents Med Chem*, *22*, 164–197. 10.2174/0118715230272152231106094727 38038014 10.2174/0118715230272152231106094727

[8] Mehbub, M. F., Yang, Q., Cheng, Y., et al. (2024). Marine sponge-derived natural products: trends and opportunities for the decade of 2011–2020. *Frontiers in Marine Science*, *11*. 10.3389/fmars.2024.1462825

[9] Xia, J., Chen, X., Li, G., et al. (2024). A Review of Sponge-Derived Diterpenes: 2009–2022. *Marine Drugs*, *22*, 447. 10.3390/md22100447 39452855 10.3390/md22100447 PMC11509224

[10] Zhou, F., Hong, Y., Liang, R., et al. (2020). Rapid printing of bio-inspired 3D tissue constructs for skin regeneration. *Biomaterials*, *258*, 120287. 10.1016/j.biomaterials.2020.120287 32847683 10.1016/j.biomaterials.2020.120287

[11] Hamoda, A. M., Fayed, B., Ashmawy, N. S., et al. (2021). Marine Sponge is a Promising Natural Source of Anti-SARS-CoV-2 Scaffold. *Frontiers in Pharmacology*, *12*. 10.3389/fphar.2021.666664 10.3389/fphar.2021.666664 PMC8165660 34079462

[12] Jimenez, P. C., Wilke, D. V., Branco, P. C., et al. (2020). Enriching cancer pharmacology with drugs of marine origin. *British Journal Of Pharmacology*, *177*, 3–27. 10.1111/bph.14876 31621891 10.1111/bph.14876 PMC6976878

[13] Fernandes, P. D., Zardo, R. S., Figueiredo, G. S. M., et al. (2014). Anti-inflammatory properties of convolutamydine A and two structural analogues. *Life Sciences*, *116*, 16–24. 10.1016/j.lfs.2014.08.019 25200874 10.1016/j.lfs.2014.08.019

[14] Di Virgilio, F., Dal Ben, D., Sarti, A. C., et al. (2017). The P2X7 Receptor in Infection and Inflammation. *Immunity*, *47*, 15–31. 10.1016/j.immuni.2017.06.020 28723547 10.1016/j.immuni.2017.06.020

[15] Kapoor, S., Nailwal, N., Kumar, M., & Barve, K. (2019). Recent Patents and Discovery of Anti-inflammatory Agents from Marine Source. *Recent Patents On Inflammation & Allergy Drug Discovery*, *13*, 105–114. 10.2174/1872213X13666190426164717 31814546 10.2174/1872213X13666190426164717

[16] Gui, Y-H., Jiao, W-H., Zhou, M., et al. (2019). Septosones A–C, in Vivo Anti-inflammatory Meroterpenoids with Rearranged Carbon Skeletons from the Marine Sponge *Dysidea septosa*. *Organic Letters*, *21*, 767–770. 10.1021/acs.orglett.8b04019 30676034 10.1021/acs.orglett.8b04019

[17] Coelho, J. F., Ferreira, P. C., Alves, P., et al. (2010). Drug delivery systems: Advanced technologies potentially applicable in personalized treatments. *EPMA Journal*, *1*, 164–209. 10.1007/s13167-010-0001-x 23199049 10.1007/s13167-010-0001-x PMC3405312

[18] Bassyouni, F., ElHalwany, N., Abdel Rehim, M., & Neyfeh, M. (2015). Advances and new technologies applied in controlled drug delivery system. *Research on Chemical Intermediates*, *41*, 2165–2200. 10.1007/s11164-013-1338-2

[19] Zhang, C., Wang, W., Zhao, X., et al. (2020). Preparation of alginate oligosaccharides and their biological activities in plants: A review. *Carbohydrate Research*, *494*, 108056. 10.1016/j.carres.2020.108056 32559511 10.1016/j.carres.2020.108056

[20] Bonifacio, M., Santi Martignago, C. C., Souza, D. C. S., et al. (2025). Chitosan Hydrogels Enriched with Biocompounds Extracted from Marine Sponges: Potential to Modulate the Inflammatory Process in an In Vitro Study. *ACS Omega*, *10*, 25605–25620. 10.1021/acsomega.5c01171 40584336 10.1021/acsomega.5c01171 PMC12199092

[21] Dash, S., Murthy, P. N., Nath, L., & Chowdhury, P. (2010). Kinetic Modeling on Drug Release from Controlled Drug Delivery Systems. *Acta Poloniae Pharmaceutica*, *67*, 217–223. 20524422

[22] Liu, S., Kang, M., Hussain, I., et al. (2016). High mechanical strength and stability of alginate hydrogel induced by neodymium ions coordination. *Polymer Degradation And Stability*, *133*, 1–7. 10.1016/j.polymdegradstab.2016.07.022

[23] Smith, A. M., & Senior, J. J. (2021). Alginate Hydrogels with Tuneable Properties. pp 37–61. 10.1007/10_2020_161 33547500

[24] Tang, C-H., Chen, L., & Foegeding, E. A. (2011). Mechanical and Water-Holding Properties and Microstructures of Soy Protein Isolate Emulsion Gels Induced by CaCl _2_, Glucono-δ-lactone (GDL), and Transglutaminase: Influence of Thermal Treatments before and/or after Emulsification. *Journal Of Agriculture And Food Chemistry*, *59*, 4071–4077. 10.1021/jf104834m 10.1021/jf104834m 21381784

[25] Lee, K. Y., & Mooney, D. J. (2012). Alginate: Properties and biomedical applications. *Progress In Polymer Science*, *37*, 106–126. 10.1016/j.progpolymsci.2011.06.003 22125349 10.1016/j.progpolymsci.2011.06.003 PMC3223967

[26] Kim, H., Song, D., Ngo, H. V., et al. (2021). Modulation of the clinically accessible gelation time using glucono-d-lactone and pyridoxal 5′-phosphate for long-acting alginate in situ forming gel injectable. *Carbohydrate Polymers*, *272*, 118453. 10.1016/j.carbpol.2021.118453 34420713 10.1016/j.carbpol.2021.118453

[27] Han, Y., Zeng, Q., Li, H., & Chang, J. (2013). The calcium silicate/alginate composite: Preparation and evaluation of its behavior as bioactive injectable hydrogels. *Acta Biomaterialia*, *9*, 9107–9117. 10.1016/j.actbio.2013.06.022 23796407 10.1016/j.actbio.2013.06.022

[28] Sun, X., Li, Z., Cui, Z., et al. (2020). Preparation and physicochemical properties of an injectable alginate-based hydrogel by the regulated release of divalent ions via the hydrolysis of < scp>d -glucono- δ -lactone. *Journal Of Biomaterials Applications*, *34*, 891–901. 10.1177/0885328219886185 31684793 10.1177/0885328219886185

[29] Costa, P., & Sousa Lobo, J. M. (2001). Modeling and comparison of dissolution profiles. *European Journal of Pharmaceutical Sciences*, *13*, 123–133. 10.1016/S0928-0987(01)00095-1 11297896 10.1016/s0928-0987(01)00095-1

[30] Wu, I. Y., Bala, S., Škalko-Basnet, N., & di Cagno, M. P. (2019). Interpreting non-linear drug diffusion data: Utilizing Korsmeyer-Peppas model to study drug release from liposomes. *European Journal of Pharmaceutical Sciences*, *138*, 105026. 10.1016/j.ejps.2019.105026 31374254 10.1016/j.ejps.2019.105026

[31] Heredia, N. S., Vizuete, K., Flores-Calero, M., et al. (2022). Comparative statistical analysis of the release kinetics models for nanoprecipitated drug delivery systems based on poly(lactic-co-glycolic acid). *PLoS One*, *17*, e0264825. 10.1371/journal.pone.0264825 35271644 10.1371/journal.pone.0264825 PMC8912140

[32] Li, C-Q., Ma, Q-Y., Gao, X-Z., et al. (2021). Research Progress in Anti-Inflammatory Bioactive Substances Derived from Marine Microorganisms, Sponges, Algae, and Corals. *Marine Drugs*, *19*, 572. 10.3390/md19100572 34677471 10.3390/md19100572 PMC8538560

[33] Scheller, J., Chalaris, A., Schmidt-Arras, D., & Rose-John, S. (2011). The pro- and anti-inflammatory properties of the cytokine interleukin-6. Biochimica et Biophysica Acta (BBA) -. *Molecular Cell Research*, *1813*, 878–888. 10.1016/j.bbamcr.2011.01.034 10.1016/j.bbamcr.2011.01.034 21296109

[34] Johnson, B. Z., Stevenson, A. W., Prêle, C. M., et al. (2020). The Role of IL-6 in Skin Fibrosis and Cutaneous Wound Healing. *Biomedicines*, *8*, 101. 10.3390/biomedicines8050101 32365896 10.3390/biomedicines8050101 PMC7277690

[35] Han, G., & Ceilley, R. (2017). Chronic Wound Healing: A Review of Current Management and Treatments. *Adv Ther*, *34*, 599–610. 10.1007/s12325-017-0478-y 28108895 10.1007/s12325-017-0478-y PMC5350204

[36] Wang, T., & He, C. (2018). Pro-inflammatory cytokines: The link between obesity and osteoarthritis. *Cytokine & Growth Factor Reviews*, *44*, 38–50. 10.1016/j.cytogfr.2018.10.002 30340925 10.1016/j.cytogfr.2018.10.002

[37] Hu, T. Y., Zhang, H., Chen, Y. Y., Jiao, W. H., Fan, J. T., Liu, Z. Q., Lin, H. W., & Cheng, B. H. (2021). Dysiarenone from marine sponge dysidea arenaria attenuates ROS and inflammation via inhibition of 5-LOX/NF-κB/MAPKs and upregulation of Nrf-2/OH-1 in RAW 264.7 Macrophages. *Journal of Inflammation Research*, *14*, 587–597. 10.2147/JIR.S283745 33664584 10.2147/JIR.S283745 PMC7921866

[38] Huong, D. T. M., Quynh, D. T., Van, T. T. T., et al. (2023). A New Alkaloid from Marine-Derived Actinomycete Actinoalloteichus cyanogriseus G631. *Records of Natural Products*, 1024–1030. 10.25135/rnp.412.2305.2776

[39] Seo, S. S., Kim, O. G., Seo, J. H., Kim, D. H., Kim, Y. G., & Park, B. Y. (2017). Comparison of the effect of continuous femoral nerve block and adductor canal block after primary total knee arthroplasty. *CiOS Clinics in Orthopedic Surgery*, *9*(3), 303–309. 10.4055/cios.2017.9.3.303 28861197 10.4055/cios.2017.9.3.303 PMC5567025

[40] Kim, B. S., Kwon, Y. W., Kong, J-S., et al. (2018). 3D cell printing of in vitro stabilized skin model and in vivo pre-vascularized skin patch using tissue-specific extracellular matrix bioink: A step towards advanced skin tissue engineering. *Biomaterials*, *168*, 38–53. 10.1016/j.biomaterials.2018.03.040 29614431 10.1016/j.biomaterials.2018.03.040

