# Supplementary material for: Analysis of the Anti-inflammatory Effects of a Delivery System Based on Alginate Hydrogels Enriched with Bioactive Compounds from Marine Sponge Dysidea robusta
Source: Appl Biochem Biotechnol. 2026 Apr 6;198(7):5023–44. doi: 10.1007/s12010-026-05684-z (PMC13287294; doi:10.1007/s12010-026-05684-z)
Supplement: Supplementary file 1 — Supplementary Material 1 [file 12010_2026_5684_MOESM1_ESM.pdf]

Supplementary Information

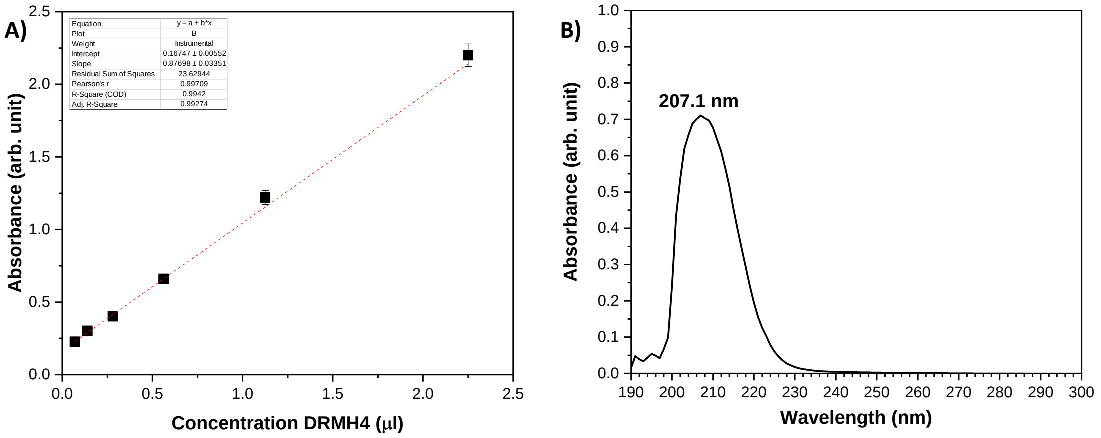

Figure S1. A) Standard calibration curve of DRMH4 fraction and **B)** respective absorption spectra of DRMH4 fraction.

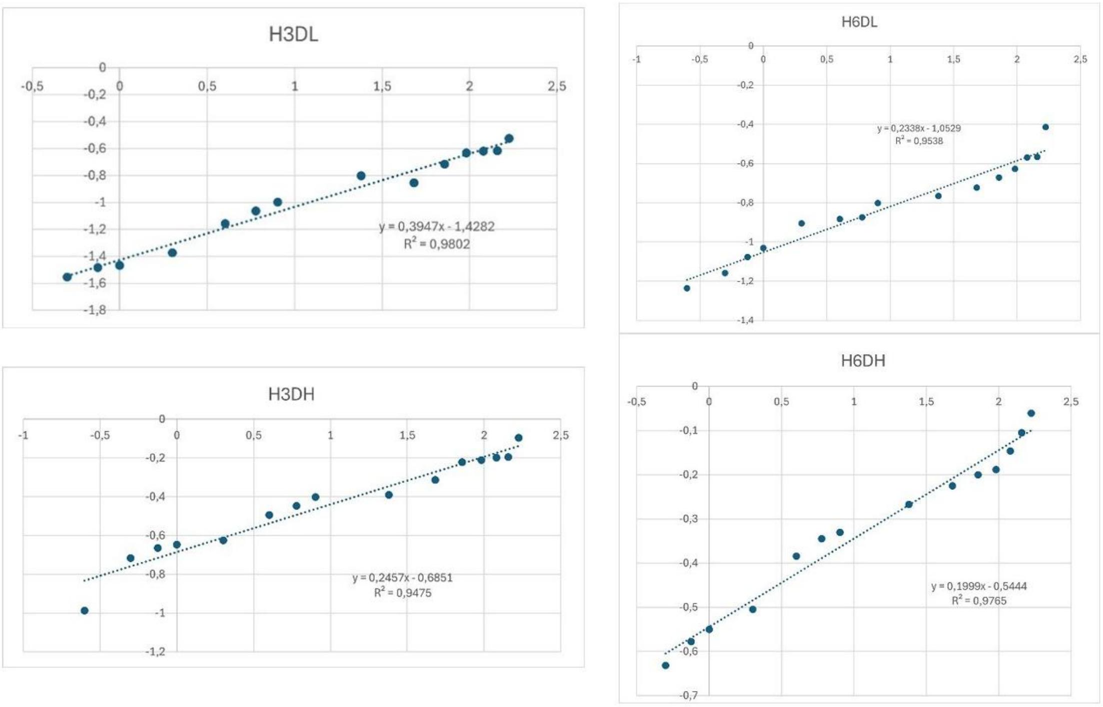

Figure S2. Non-linear data fitting using the Korsmeyer-Peppas model
