# Supplementary material for: Analysis of the Anti-inflammatory Effects of a Delivery System Based on Alginate Hydrogels Enriched with Bioactive Compounds from Marine Sponge Dysidea robusta
Source: Appl Biochem Biotechnol. 2026 Apr 6;198(7):5023–44. doi: 10.1007/s12010-026-05684-z (PMC13287294; doi:10.1007/s12010-026-05684-z)
Supplement: Supplementary file 2 — Supplementary Material 2 [file 12010_2026_5684_MOESM2_ESM.pdf]

August 21st, 2025

Dear Editor,

Please find attached the manuscript "Analysis of the anti-inflammatory effects of a delivery system based on alginate hydrogels enriched with bioactive compounds from marine sponge *Dysidea robusta*".

Please Consider the following statements:

1. The article is original;
2. The article has been written by the stated authors who are ALL aware of its content and approve its submission;
3. The article has not been published previously;
4. The article is not under consideration for publication elsewhere;
5. No conflict of interest exists, or if such conflict exists, the exact nature must be declared.
6. If accepted, the article will not be published elsewhere in the same form, in any language, without the written consent of the publisher.

We would also like to request a fee waiver due to the partnership between ACS and the Fundação Coordenação de Aperfeiçoamento de Pessoal de Nível Superior (CAPES).

In short, we appreciate your considering our submission and look forward to your feedback. Please feel free to contact me with any questions or for more information.

Best regards,

PhD. Ana Claudia Muniz Rennó
(On behalf of all authors).

Departamento de Biociências - UNIFESP
Rua Silva Jardim 136, Edifício Central, Santos, SP, Brazil
Email: amanda_desouza@outlook.com
